# Deletion of *Tbc1d4*/As160 abrogates cardiac glucose uptake and increases myocardial damage after ischemia/reperfusion

**DOI:** 10.1186/s12933-023-01746-2

**Published:** 2023-01-27

**Authors:** C. Binsch, D. M. Barbosa, G. Hansen-Dille, M. Hubert, S. M. Hodge, M. Kolasa, K. Jeruschke, J. Weiß, C. Springer, S. Gorressen, J. W. Fischer, M. Lienhard, R. Herwig, S. Börno, B. Timmermann, A. L. Cremer, H. Backes, A. Chadt, H. Al-Hasani

**Affiliations:** 1 Medical Faculty, Institute for Clinical Biochemistry and Pathobiochemistry, German Diabetes Center, Leibniz-Center for Diabetes Research at Heinrich Heine University Düsseldorf, Auf’m Hennekamp 65, 40225 Düsseldorf, Germany; 2 Institute for Pharmacology and Clinical Pharmacology, Heinrich-Heine-University, Düsseldorf, Germany; 3 Max-Planck-Institute for Molecular Genetics, Berlin, Germany; 4 Max Planck Institute for Metabolism Research, Cologne, Germany; 5 German Center for Diabetes Research, Partner Düsseldorf, Munich-Neuherberg, Germany

**Keywords:** Myocardial infarction, Ischemia/reperfusion, TBC1D4, Metabolic flexibility

## Abstract

**Background:**

Type 2 Diabetes mellitus (T2DM) is a major risk factor for cardiovascular disease and associated with poor outcome after myocardial infarction (MI). In T2DM, cardiac metabolic flexibility, i.e. the switch between carbohydrates and lipids as energy source, is disturbed. The RabGTPase-activating protein TBC1D4 represents a crucial regulator of insulin-stimulated glucose uptake in skeletal muscle by controlling glucose transporter GLUT4 translocation. A human loss-of-function mutation in TBC1D4 is associated with impaired glycemic control and elevated T2DM risk. The study’s aim was to investigate TBC1D4 function in cardiac substrate metabolism and adaptation to MI.

**Methods:**

Cardiac glucose metabolism of male *Tbc1d4*-deficient (D4KO) and wild type (WT) mice was characterized using in vivo [^18^F]-FDG PET imaging after glucose injection and ex vivo basal/insulin-stimulated [^3^H]-2-deoxyglucose uptake in left ventricular (LV) papillary muscle. Mice were subjected to cardiac ischemia/reperfusion (I/R). Heart structure and function were analyzed until 3 weeks *post-*MI using echocardiography, morphometric and ultrastructural analysis of heart sections, complemented by whole heart transcriptome and protein measurements.

**Results:**

*Tbc1d4-*knockout abolished insulin-stimulated glucose uptake in ex vivo LV papillary muscle and in vivo cardiac glucose uptake after glucose injection, accompanied by a marked reduction of GLUT4. Basal cardiac glucose uptake and GLUT1 abundance were not changed compared to WT controls. D4KO mice showed mild impairments in glycemia but normal cardiac function. However, after I/R D4KO mice showed progressively increased LV endsystolic volume and substantially increased infarction area compared to WT controls. Cardiac transcriptome analysis revealed upregulation of the unfolded protein response via ATF4/eIF2α in D4KO mice at baseline. Transmission electron microscopy revealed largely increased extracellular matrix (ECM) area, in line with decreased cardiac expression of matrix metalloproteinases of D4KO mice.

**Conclusions:**

TBC1D4 is essential for insulin-stimulated cardiac glucose uptake and metabolic flexibility. *Tbc1d4*-deficiency results in elevated cardiac endoplasmic reticulum (ER)-stress response, increased deposition of ECM and aggravated cardiac damage following MI. Hence, impaired TBC1D4 signaling contributes to poor outcome after MI.

**Supplementary Information:**

The online version contains supplementary material available at 10.1186/s12933-023-01746-2.

## Background

Type 2 diabetes mellitus (T2DM) constitutes a major risk factor for the development of cardiovascular disease (CVD) [1, 2]. Individuals with T2DM exhibit increased prevalence for myocardial infarction (MI), impaired recovery and reduced survival rates after infarction [3]. The incidence of CVD in T2DM patients is estimated to be two to eight-fold higher compared to healthy individuals [4]. Moreover, in the state of diabetic cardiomyopathy, myocardial function and structure can be impaired in the absence of additional cardiac risk factors, like hypertension or coronary artery disease [5].

A key feature of the healthy heart is the high metabolic flexibility, which ensures optimal adaptation of energy provision to substrate availability and energy requirements (e.g. during resting, exercise). This flexibility is achieved by finely regulating the utilization of lipids, glucose and other substrates for ATP generation [6, 7]. Under healthy, well-perfused conditions, the major part (60–90%) of the hearts’ energy production is provided by lipids, whereas glucose and lactate only constitute a minor proportion of 10–40% in the total substrate use [8], next to a lesser portion derived from ketone bodies and amino acids that can be utilized for energy production in addition by cardiomyocytes [9, 10]. In situations of stress and increased energy demand, such as exercise or ischemia, the substrate preference of the heart is shifted towards the predominant use of glucose [11] in order to ensure sufficient cardiac energy supply. In T2DM, this metabolic flexibility is disturbed due to an imbalance of glucose and lipid utilization, manifesting in a reduced uptake and utilization of glucose along with an increased oxidation of lipids [12].

Thus, while under normal conditions, fatty acids are the main energy source of the heart, knockdown and overexpression studies of the two predominant glucose transporters GLUT4 and GLUT1 in rodents indicate that cardiac glucose uptake is essential for heart function and survival, in particular after hypoxic stress [11].

The two related ~ 160 kDa Rab-GTPase activating proteins (RabGAPs) TBC1D1 and TBC1D4 are downstream targets of AKT and AMPK and play key roles in regulating insulin-stimulated glucose uptake in skeletal muscle and adipose cells by mediating translocation of glucose transporter type 4 (GLUT4) from cytosolic storage vesicles to the plasma membrane [13, 14]. Especially in skeletal muscle, TBC1D1 is the predominant form in glycolytic fiber types whereas TBC1D4 was shown to be mainly expressed in oxidative muscle [15].

We and others previously demonstrated that *Tbc1d4*-deficient mice present markedly reduced insulin-stimulated glucose uptake into skeletal muscle and adipose cells, associated with reduced abundance of GLUT4 protein [15, 16]. Mice lacking *Tbc1d4* [16, 17] showed rather mild impairments in whole-body glycemic control whereas deletion of both RabGAPs augmented the insulin resistance, indicative of redundancy in insulin signaling [16, 17].

A common muscle-specific *TBC1D4* p.Arg684Ter loss-of-function variant has been identified previously in arctic populations as a major contributor to the development of insulin resistance and T2DM [18, 19]. While TBC1D4 is also expressed in the heart [16, 17], the impact of RabGAP deficiency on cardiac metabolism has not yet been investigated in this issue. Interestingly, a recent study found that homozygous carriers of the p.Arg684Ter variant carry an increased risk for CVD, ischemic heart disease and CVD-related death but failed to demonstrate statistical significance in Greenlandic Inuit [19].

Here, we speculate that TBC1D4 is not only an important regulator of glucose and lipid metabolism in striated muscle cells but also cardiac glucose utilization. Moreover, we aim to show that TBC1D4-mediated changes in cardiac substrate utilization are directly linked to ischemia/reperfusion-induced injury after MI.

## Material and methods

### Experimental animals

Male mice with targeted whole-body deletion of *Tbc1d4* (D4KO) on a C57BL/6J background were generated as described [15]. After weaning at 19–21 days of age, animals received a standard chow with 19% (wt/wt) protein (23 cal%), 3.3% fat (8 cal%), and 54.1% carbohydrates (69 cal%) containing 3.06 kcal/g energy (V153 3 R/M-H; Ssniff, Soest, Germany) or a high-fat diet (HFD) with 26.2% (wt/wt) protein (20 cal%), 34.9% fat (60 cal%), and 26.3% carbohydrates (60 cal%) containing 5.24 kcal/g energy (D12492; Research Diets Inc. New Brunswick, NJ, USA), respectively. If not stated otherwise, all in vivo experimental procedures as well as tissue collection were conducted between 8 a.m. and 11 a.m. with mice in the random fed state.

### Genotyping

Isolation of DNA from mouse tail tips was performed using InViSorb Genomic DNA Kit II (Stratec, Birkenfeld, Germany). Mice were genotyped via PCR using three primers for the *Tbc1d4*-knockout allele (Fwd: 5’-AGTAGACTCAGAGTGGTCTTGG-3’; Rev-WT: 5’-GTCTTCCGACTCCATATTTGC-3’; Rev-KO: 5’-GCAGCGCATCGCCTTCTATC-3’).

### Ischemia/Reperfusion surgery

At 36 weeks of age, HFD-fed mice were subjected to cardiac ischemia/reperfusion operations in a closed chest and open chest model, respectively.

#### Open chest

Mice were anesthetized via an *intraperitoneal* injection with Ketamine (100 mg/kg body weight) and Xylazine (10 mg/kg body weight), intubated and fixated on a pre-heated (37.5 °C) surface. Constant respiration was applied with O_2_- enriched (40% O_2_) air and 2% Isoflurane. Mice were constantly monitored via electrocardiography during the operation and maintained body temperature during the whole procedure at 37–38 °C. Subsequently, the thorax was opened via lateral thoracotomy and cardiac ischemia was performed by ligation of the left anterior descending (LAD) coronary artery. A ligature was placed around the LAD with a suture and reversibly occluded using a piece of polyethylene tubing. Occlusion was ensured via visible paling of the proximal cardiac tissue and elevation of ST segment of ECG. Ischemia was maintained for 45 min. After this time, the suture was removed and the thorax was closed with sutures. Isoflurane application was terminated and mice were ventilated for some more min and subsequently extubated. During the following 5 days mice were treated with buprenorphine (0.05 mg/kg body weight) every 6 h.

#### Closed chest

The LAD was surrounded with a suture and a piece of polyethylene tube, but not occluded. The ends of the suture were removed from the thorax and placed under the skin with a knot. Thorax and skin were closed. Isoflurane application was terminated and mouse rested for 3 days with close observation. For post-operative treatment, mice were subcutaneously injected with buprenorphine (0.05 mg/kg body weight) every 6 h. Three days after the ligature, mice were anesthetized with oxygen enriched air (40% O_2_) and 2% Isoflurane. Under constant ECG and temperature monitoring, skin was incised and ischemia was induced by closing of the ligature by pulling at the end of the sutures. After 60 min of ischemia, sutures were cut and skin closed. Isoflurane was removed and mice were ventilated for some more minutes and subsequently extubated. During the following 5 days mice were treated with buprenorphine (0.05 mg/kg body weight) every 6 h.

### Ex vivo glucose uptake by left ventricular papillary muscle

[^3^H]-2-deoxyglucose uptake in intact isolated left ventricular papillary muscle was essentially performed as previously described for skeletal muscle incubations [20]. Some modifications of the protocol were made in order to account for the different tissue type. Briefly, after 4 h of fasting mice were injected with 100U Heparin, subsequently euthanized via cervical dislocation and LV papillary muscles were dissected in pre-oxygenated (95% oxygen/5% carbon dioxide) Krebs–Henseleit buffer (KHB) (118.5 mM NaCl, 4.7 mM KCl, 1.2 mM KH_2_PO_4_, 25 mM NaHCO_3_, 4.7 mM KCl, 2.5 mM CaCl_2_ · 2H_2_O, 1.2 mM MgSO_4_ 7HO, 5 mM HEPES, 1% BSA) supplemented with 5 mmol/L glucose and 15 mmol/L mannitol and incubated for 30 min at 30 °C in vials containing pre-oxygenated (95% oxygen/5% carbon dioxide) KHB supplemented with 5 mmol/L glucose and 15 mmol/L mannitol. All incubation steps were conducted under continuous gas supply (95% oxygen/5% carbon dioxide) at 30 °C and gentle agitation in a water bath. After recovery, muscles were transferred to new vials and incubated for 30 min in KHB supplemented with 15 mmol/L mannitol and 5 mmol/L glucose under basal conditions or with 120 nmol/L insulin throughout the duration of the experiment. Consequently, muscles were incubated for 10 min in KHB containing 20 mmol/L mannitol under basal conditions or in the presence of 120 nmol/L insulin before being transferred to the radioactive glucose transport incubation step. After 20 min of incubation in the presence of 1 mmol/L [^3^H]-2-deoxyglucose and 19 mol/L [^14^C]mannitol, muscles were immediately frozen in liquid nitrogen and stored at − 80 °C. Cleared protein lysates were used to determine incorporated radioactivity by scintillation counting. [^14^C] counts from mannitol were measured as background control to correct for the ECM-bound partition of [^3^H]-2-deoxyglucose not transported into the cells.

### Echocardiography

Echocardiography was performed using a Vevo 2100 high-resolution ultrasound scanner with 18 to 38 MHz linear transducer (VisualSonics, Inc) as previously described [21], before MI and at time points of 24 h, 1 week and 3 weeks after reperfusion, respectively. Parameters of LV end-systolic and end-diastolic volumes were measured.

### Quantitative real-time-PCR (qRT-PCR)

RNA was isolated and cDNA was synthesized as previously described [20]. Quantitative Real-time PCR (qRT-PCR) was performed using a 7500 Fast Real-Time PCR System with SYBR Green (Applied Biosystems, Foster City, CA) and suitable PCR primers for *Tbc1d1, Tbc1d4, Slc2a1, Slc2a4*, *Atf4* as well as spliced and unspliced variants of *Xbp*. Data was normalized to *Tbp* expression according to the ΔCt method [22].

Relative copy number of cardiac mRNA of *Tbc1d1* and *Tbc1d4* was assessed via normalized ΔCt values using a calibration curve obtained from the amplification of plasmids containing the respective cDNA sequences as previously described [23].

### Western blot analysis

Tissues were homogenized in lysis buffer (20 mmol/L Tris, 150 mmol/L NaCl, 1 mmol/L EGTA, 1 mmol/L EDTA, 1% [v/v] Triton-X-100, and both, a proteinase inhibitor and a phosphatase inhibitor cocktail; Complete and PhosSTOP; Roche, Mannheim, Germany) and centrifuged for 10 min at 16,000 relative centrifugal force at 4 °C. Protein content of the supernatant was determined using the BCA Protein Assay Kit (Pierce, Rockford, IL, USA). Immunoblotting and detection was performed with an ECL Western blot detection analysis system (GE Healthcare, Buckinghamshire, UK), as described previously [15]. Primary antibody suppliers and Western Blotting conditions are listed in Additional file 8: Table S2.

### RNA Sequencing and transcriptome analysis

RNA was isolated using the miRNeasy kit (Qiagen, Hilden, Germany) according to the manufacturer’s instructions. Sequencing libraries were prepared from polyA selected mRNA and sequenced on Illumina HiSeq2500 platform, yielding 25.5 to 82.6 million 2 × 75 base read pairs per sample (n = 4).

RNASeq reads were aligned with STAR v2.4.1d and 75% to 85% of the reads mapped to a unique genomic position. Reads were counted per gene, using htseq-count version 0.6.1p1 in “union” mode. For normalization and detection of differentially expressed genes, the Bioconductor package DESeq2 was used. In brief, read counts per gene were imported using DESeq2s’ DESeqDataSetFromHTSeqCount function. After estimating size factors and dispersion parameters, we applied the negative binomial Wald test. Genes with adjusted p-value < 0.01 were considered differentially expressed and exported for enrichment pathway analysis.

Enrichment and canonical pathway analyses as well as upstream target analyses were performed using Ingenuity pathway analysis software (Qiagen, Hilden, Germany) and ConsensusPathDB [24]. Cut-offs were set according to an adjusted p-value < 0.01 for all analyses.

### Histomorphometry

Mouse heart was treated with heparin and subsequently fixed in 4% PFA/PBS, pH 7.4 overnight at 4° C. Samples were then processed as paraffin blocks and 5 µM sections were stained with Azan. For morphometric evaluation of Azan-stained connective tissue after Ischemia/Reperfusion, threshold analysis was used.

### Transmission electron microscopy

Heart muscle tissues were fixed overnight at 4 °C by immersion in 2.5% glutaraldehyde in 0.19 M sodium cacodylate buffer at pH 7.4, postfixed in 1% reduced osmium tetroxide in aqua bidest for 90 min, and subsequently stained with 2% uranyl acetate in maleate buffer, pH 4.7. The specimens were dehydrated in graded ethanols and embedded in epoxy resin [25]. Ultrathin sections were picked up onto Formvarcarbon-coated grids, stained with lead citrate [26], and viewed in a transmission electron microscope (TEM 910; Zeiss Elektronenmikroskopie, Oberkochen, Germany).

Morphometric evaluation of extracellular matrix area (ECM) was done using comparable ROI (regions of interest) of heart muscle sections excluding cellular components inside the ECM area. For evaluation threshold analysis was applied.

### PET data acquisition

[^18^F]-FDG PET data were acquired using a combined preclinical PET/CT scanner (Inveon, Siemens). For the measurement, animals were placed on a water-heated mouse carrier (Medres) and anesthetized with ~ 2% isoflurane in a 70% nitrous oxide/30% oxygen gas mixture. For injection of the radiotracer, a catheter consisting of a cannula connected to a polythene tubing was inserted into the tail vein of each mouse. At the start of the 45 min PET data acquisition, the animals received an injection of 10 µCi/g(BW) [^18^F]-FDG mixed with 1 mg/g(BW) glucose via the tail vein. Following the PET scan the animals were automatically moved into the CT gantry and a CT scan was performed (180 projections/360°, 200 ms, 80 kV, 500 μA) with the whole mouse in the field of view. CT data were used for attenuation correction of the PET data. PET data were histogrammed in 25 time frames of 12 × 30 s, 3 × 60 s, 3 × 120 s and 7 × 240 s, rebinned in 3D and after correction for attenuation and decay, images were reconstructed using the MAP-SP algorithm provided by the manufacturer.

Image analysis was performed using the VINCI software (VINCI 4.90, MPI for Metabolism Research). For the analysis of glucose uptake into the heart, a volume of interest (VOI) containing the entire heart was defined for each individual animal. Whole-body activity was calculated as the average activity of the last 20 min of the PET scan in a VOI containing the whole mouse. To account for the [^18^F]-FDG that is not recycled in the kidney due to the low efficiency of the SGLT1 to transport [^18^F]-FDG, we subtracted the total activity in kidney and bladder from the whole-body activity at each time point. The total activity in the heart was normalized to this corrected whole-body activity at each time point.

### Statistics

Statistical analysis was performed using GraphPad Prism 7 software. Data are reported as mean ± SEM. Significant differences were determined by one-way or two-way ANOVA (post-hoc-test, Bonferroni multiple comparison test) or paired two-tailed Student’s t-test, as indicated in the figure legends. P-values < 0.05 were considered statistically significant.

## Results

### Deficiency of *Tbc1d4* abolishes insulin-stimulated glucose uptake in LV papillary muscle in vitro and reduces cardiac GLUT4 abundance

**Fig. 1:**
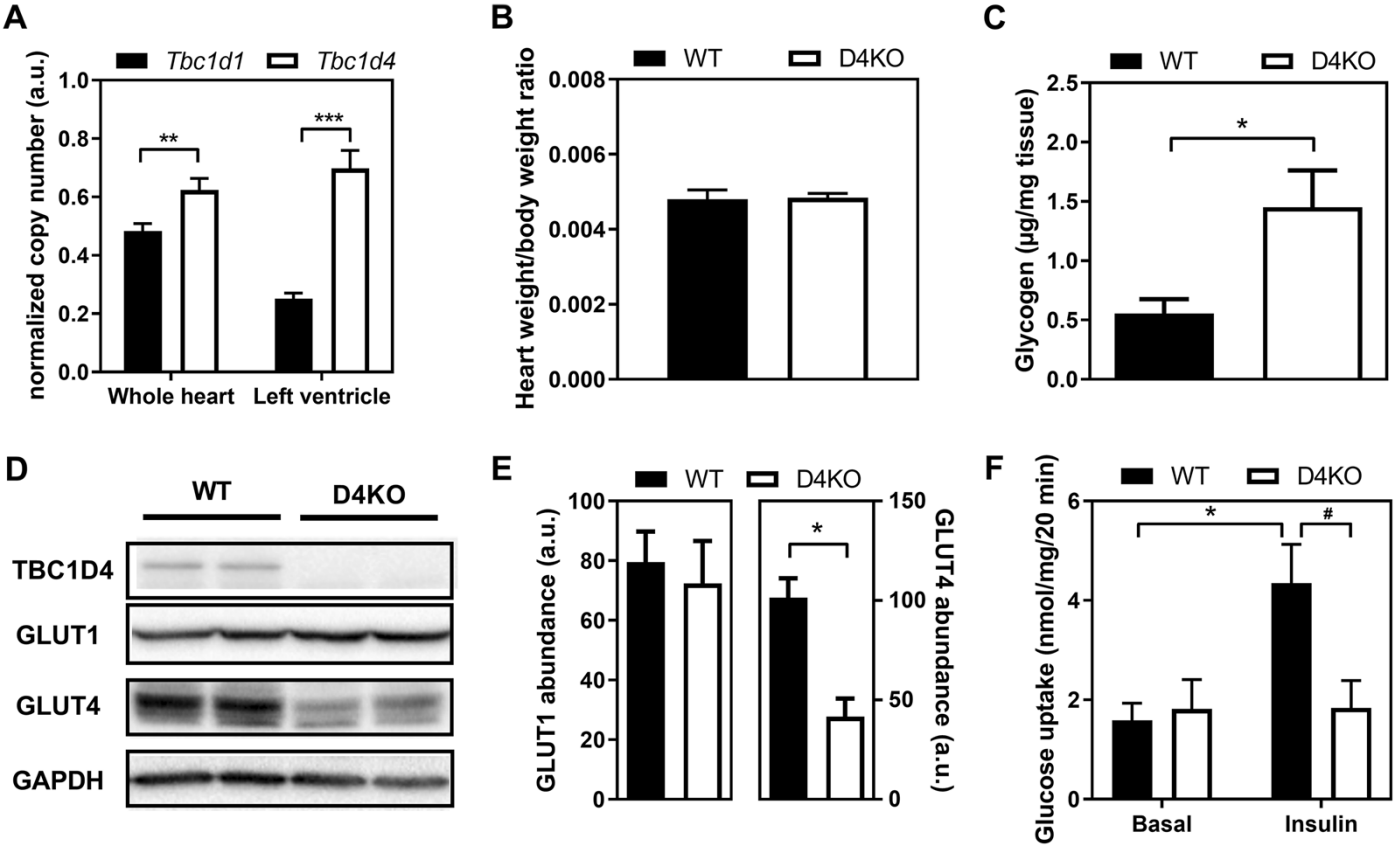
Deletion of *Tbc1d4* leads to reduced cardiac GLUT4 content and impaired ex vivo glucose uptake. **A** mRNA copy number, normalized by *Tbp* expression, of *Tbc1d1* and *Tbc1d4* in whole heart tissue and isolated left ventricle. **B** Heart weight/body weight ratio of WT and D4KO animals on standard diet. **C** Total cardiac glycogen content (n = 7–8); **D**, **E** Cardiac protein abundance of TBC1D4, GLUT1 and GLUT4 (n = 7), **F** Insulin-stimulated [^3^H]-2-deoxyglucose uptake in isolated cardiac left-ventricular papillary muscle (n = 5–6). Data presented as mean ± SEM. Statistical analysis was performed using unpaired two-tailed student’s t-Test (A-C,E; WT vs. D4KO: *p < 0.05; **p < 0.01; ***p < 0.001) or two-way ANOVA with Bonferroni’s multiple comparison test (F; basal vs. insulin: *p < 0.05; WT vs. D4KO: #p < 0.05); male mice, 36 weeks of age. *WT* wild type, *D4KO* *Tbc1d4*-knockout

We determined cardiac mRNA copy numbers of both *Tbc1d1* and *Tbc1d4* by quantitative real-time PCR as described before [23]. In C57BL/6 J mice, *Tbc1d4* represents the major RabGAP isoform expressed in the heart when compared to its paralogue *Tbc1d1*, especially in the cardiac left ventricle (Fig. 1A). Exon-specific PCR analysis revealed that heart tissue mainly contains the long 1298 aa isoform of *Tbc1d4*, similar to skeletal muscle (Additional file 1: Figure S1).

Previously, we and others demonstrated that *Tbc1d4*-deficient (D4KO) mice exhibit reduced insulin-stimulated glucose uptake in oxidative skeletal muscle and adipose cells but maintain euglycemia [15]. As illustrated in Fig. 1B, D4KO mice showed no difference in heart weight-to-body weight ratio when compared to WT littermates. However, glycogen content was increased in total heart tissue of D4KO animals compared to WT controls (Fig. 1C). Glycogen phosphorylase (PYGB) abundance was not changed (data not shown). While GLUT4 protein was substantially reduced in D4KO hearts, the abundance of cardiac GLUT1 protein remained unchanged compared to WT animals (Fig. 1D, 1E). We next determined basal and insulin-stimulated glucose uptake in intact isolated papillary muscle as described in the methods section. Accordingly to the glucose transporter abundance, basal [^3^H]-2-deoxyglucose uptake in isolated intact left ventricular papillary muscle was not different between the genotypes. In contrast, insulin-stimulated glucose uptake was abolished in isolated papillary muscle of D4KO mice compared to WT littermates (Fig. 1F). Moreover, uptake of [^3^H]Palmitate into papillary muscle ex vivo and cardiac expression and abundance of fatty acid transporter proteins CD36/FAT, FATP4 and FATP6 was not different between the genotypes (Additional file 5: Figure S5).

### Deficiency of *Tbc1d4* abolishes in vivo glucose uptake into the heart after glucose infusion

**Fig. 2:**
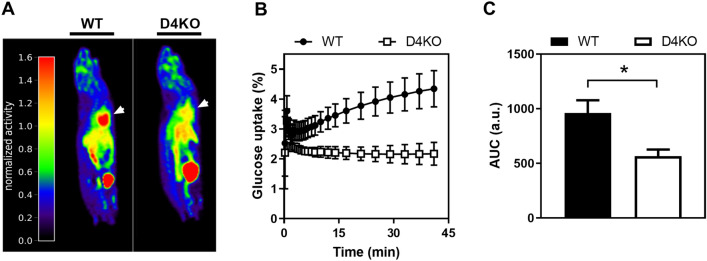
Deletion of *Tbc1d4* leads to impaired in vivo cardiac glucose uptake. **A** [^18^F]-fluorodeoxy-D-glucose positron emission tomography (PET) scan technique was applied in order to visualize glucose uptake into the heart (white arrow) for male Tbc1d4-deficient and wild type mice on a Chow-diet. **B** Quantification of cardiac glucose uptake over time with corresponding (C) area under the curve (AUC) calculation. Data presented as mean ± SEM (n = 4). Statistical analysis was performed using unpaired two-tailed student’s t-Test (C; WT vs. D4KO: *p < 0.05). *WT* wild type, *D4KO* *Tbc1d4*-knockout

For investigating glucose flux into the heart in vivo, we employed [^18^F]-fluorodeoxy-D-glucose (FDG) positron emission tomography combined with computed tomography (FDG-PET/CT). As described in the methods section, D4KO mice and WT littermates were administered an *i.v.* glucose bolus with tracer FDG into the tail vein, followed by PET and CT scans. Consistent with the ex vivo measurements of insulin-stimulated glucose uptake into cardiac muscle, glucose uptake into the hearts of D4KO animals was completely blunted whereas WT mice displayed a substantial increase in FDG tracer accumulation after glucose infusion (Fig. 2A–C). This effect was not only observed during the whole measurement process, but also within the first 5 min after glucose infusion (Additional file 4: Figure S4). The glucose uptake of the brain was not altered between D4KO and WT animals (Additional file 6: Figure S6).

### Deficiency of *Tbc1d4* under HFD aggravates ischemia/reperfusion injury following myocardial infarction

**Fig. 3:**
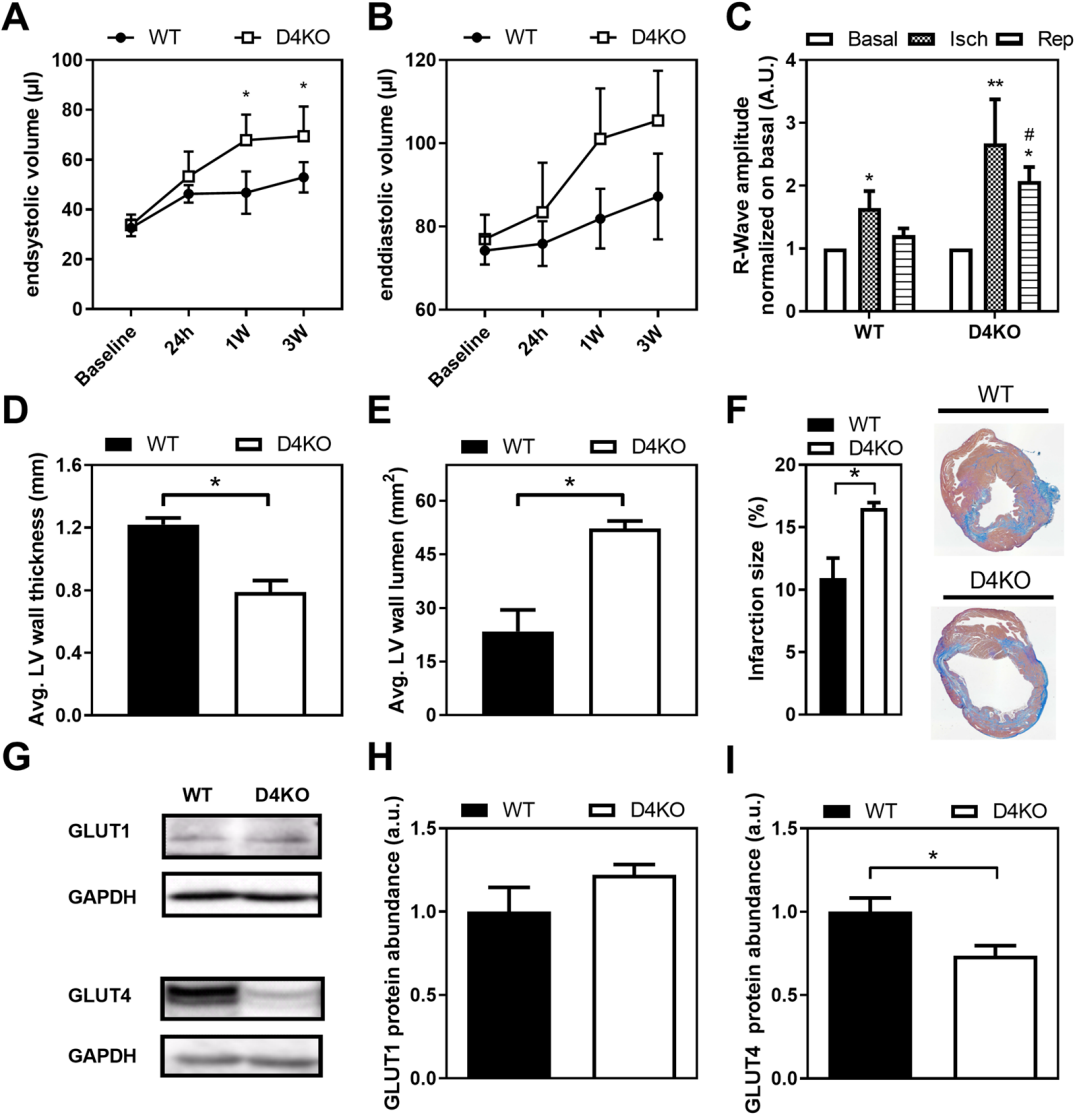
*Tbc1d4*-ko and HFD impair heart function and morphology following I/R with unaffected reduced GLUT4 content. Echocardiographic assessment of cardiac **A** end-systolic and **B** end-diastolic volume over a time course of 3 weeks (n = 6). **C** R-wave amplitude at basal state, during ischemia and acute reperfusion phase, normalized to the basal state. Histomorphometrical analysis of WT and *Tbc1d4*-deficient hearts 3 weeks post-I/R (closed chest) in terms of: **D** left ventricular wall thickness, **E** left ventricular lumen, and **F** infarct size (n = 3). **G**, **H**, **I** Cardiac protein content of **H** GLUT1 and **I** GLUT4 three weeks after reperfusion (open chest; n = 6–7). Data presented as mean ± SEM. Statistical analysis was performed using unpaired two-tailed student’s t-Test (A, B, D-F, H, I; WT vs. D4KO: *p < 0.05) or two-way ANOVA with Bonferroni’s multiple comparison test (C; *Isch* or *Rep* vs. basal: *p < 0.05, **p < 0.01; WT vs. D4KO: #p < 0.05) (Male mice, 36 weeks of age). *WT* wild type, *D4KO* *Tbc1d4*-knockout, *Isch* ischemia, *Rep* Reperfusion

Next, the impact of *Tbc1d4* knockout on cardiac function in vivo and the response to myocardial infarction were assessed. We induced a mild glucose intolerance by feeding the animals a HFD (Additional file 2: Figure S2). Subsequently, we generated ischemia/reperfusion (I/R) injury using a closed chest model to induce ischemia (60 min) by occlusion of the left anterior descending (LAD) artery, following reperfusion as described in the methods section. At baseline before the intervention, heart function of WT and D4KO mice under high-fat diet conditions was not different as assessed by echocardiography. However, echocardiographic analysis over a time course of 3 weeks following the reperfusion revealed a progressive increase in end-systolic volume (Fig. 3A) and a tendency towards increased end-diastolic volume (Fig. 3B) in D4KO animals compared to WT controls (cf. Additional file 3: Figures S3 and Additional file 7: Figure S7). During monitoring of the increase of the cardiac R-wave amplitude in the electrocardiogram as control for accurate ligation, we observed that an increase in the R-wave amplitude of D4KO animals relative to the baseline in the acute reperfusion phase in when compared to the WT situation (Fig. 3C). Three weeks *post* reperfusion, hearts were harvested from D4KO mice and WT littermates. Subsequently, histological sections were stained and analyzed morphometrically as described in the method section. D4KO mice showed an impaired heart structure, manifested in a substantial reduction in left ventricular wall thickness (Fig. 3D) along with a two-fold increase in left ventricular lumen (Fig. 3E). Importantly, the relative infarction size was increased by ~ 30% in D4KO mice 3 weeks *post* reperfusion (Fig. 3F). Western Blot analysis revealed that cardiac protein abundance of the glucose transporter GLUT1 was not different in D4KO and WT mice 3 weeks *post* I/R. However, GLUT4 abundance was markedly reduced by 60% in the hearts of D4KO animals (Fig. 3G–I). In order to rule out potential global impacts of high-fat diet feeding on cardiac and overall metabolism, we continued to investigate the basal effect of the *Tbc1d4*-knockout on mice fed a standard diet.

### Loss of *Tbc1d4* alters extracellular matrix morphology in the heart

**Fig. 4:**
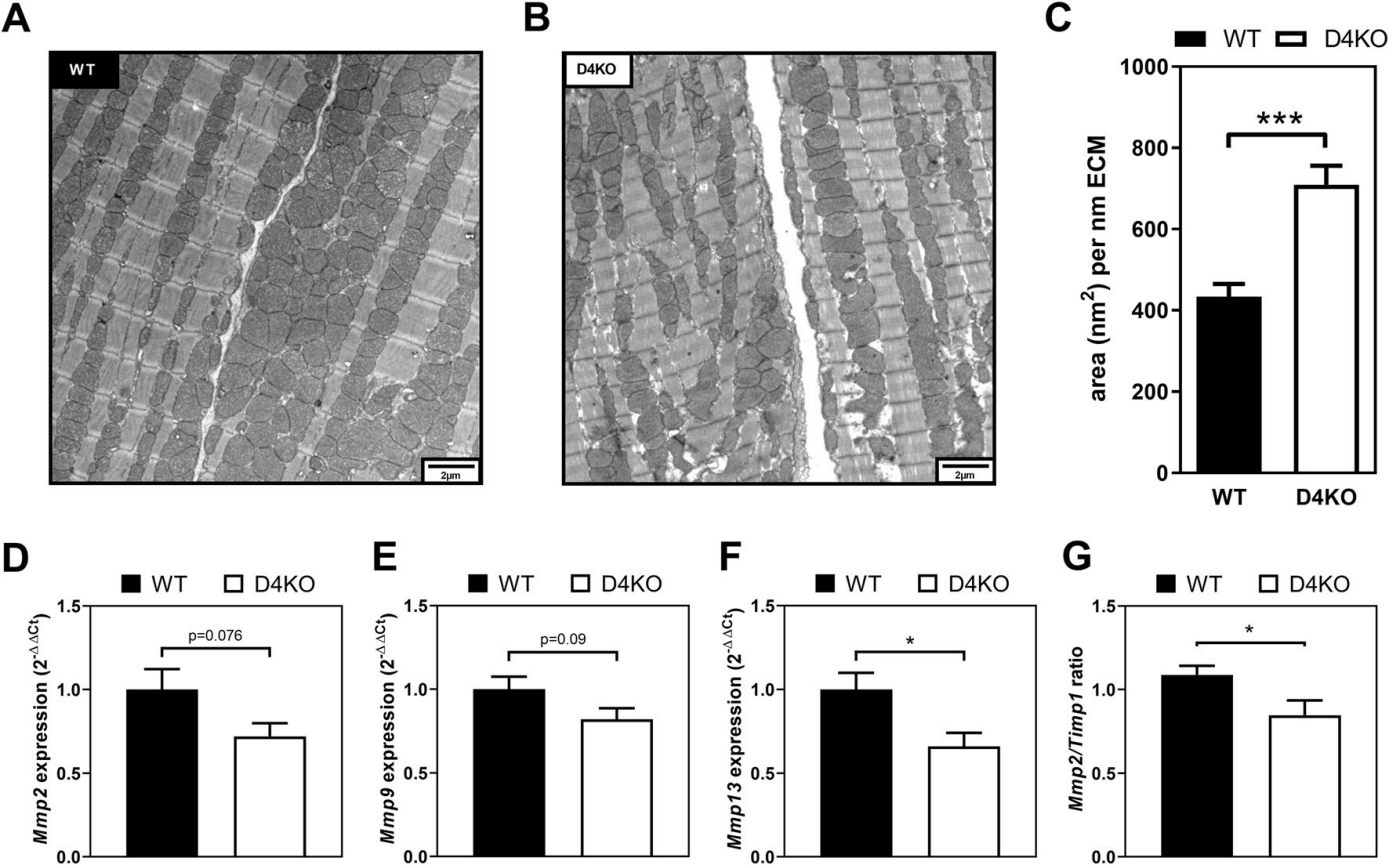
Deletion of *Tbc1d4* leads to increased extracellular matrix area and impaired *Mmp* expression profile. Transmission electron microscopy of **A** WT and **B** *Tbc1d4*-deficient cardiac left ventricular tissue and **C** histomorphometrical analysis of extracellular matrix area. Expression of ECM remodeling markers in terms of **D** *Mmp2*, **E** *Mmp9*, **F** *Mmp13* and **G** *Mmp2/Timp1* ratio. Data presented as mean ± SEM (n = 3). Statistical analysis was performed using unpaired two-tailed student’s t-Test (C-G; WT vs. D4KO: *p < 0.05; ***p < 0.001). *WT* wild type, *D4KO* *Tbc1d4*-knockout

We further investigated the morphology of hearts from D4KO mice and WT littermates at baseline conditions at the ultrastructural level by conducting transmission electron microscopy (TEM) of ultrathin sections from the left ventricle of WT and D4KO hearts. While morphometric analysis revealed no changes in cellular ultrastructure and mitochondrial size and number (data not shown), we found a robust increase in the extracellular matrix (ECM) surface area in D4KO heart tissue compared to WT littermates (Fig. 4A, B). Morphometric quantitation revealed that throughout the left ventricle, the ECM area was increased by ~ 1.5-fold in the knockout mice compared to the controls (Fig. 4C).

In line with the observed altered cardiac ultrastructure, expression of matrix metalloproteases (MMPs) and their inhibitors (TIMPs) as markers for ECM remodeling was changed in D4KO hearts as determined in qPCR experiments. Specifically, the ratio of *Mmp2*/*Timp1* as indicator for MMP action and ECM remodeling activity, and the expression of *Mmp13* were decreased in D4KO hearts, while *Mmp2* and *Mmp9* expression showed a similar trend (Fig. 4D–G).

### Deficiency of *Tbc1d4* alters cardiac transcriptome and impairs distinct pathways related to cardiac ER-stress response

**Fig. 5:**
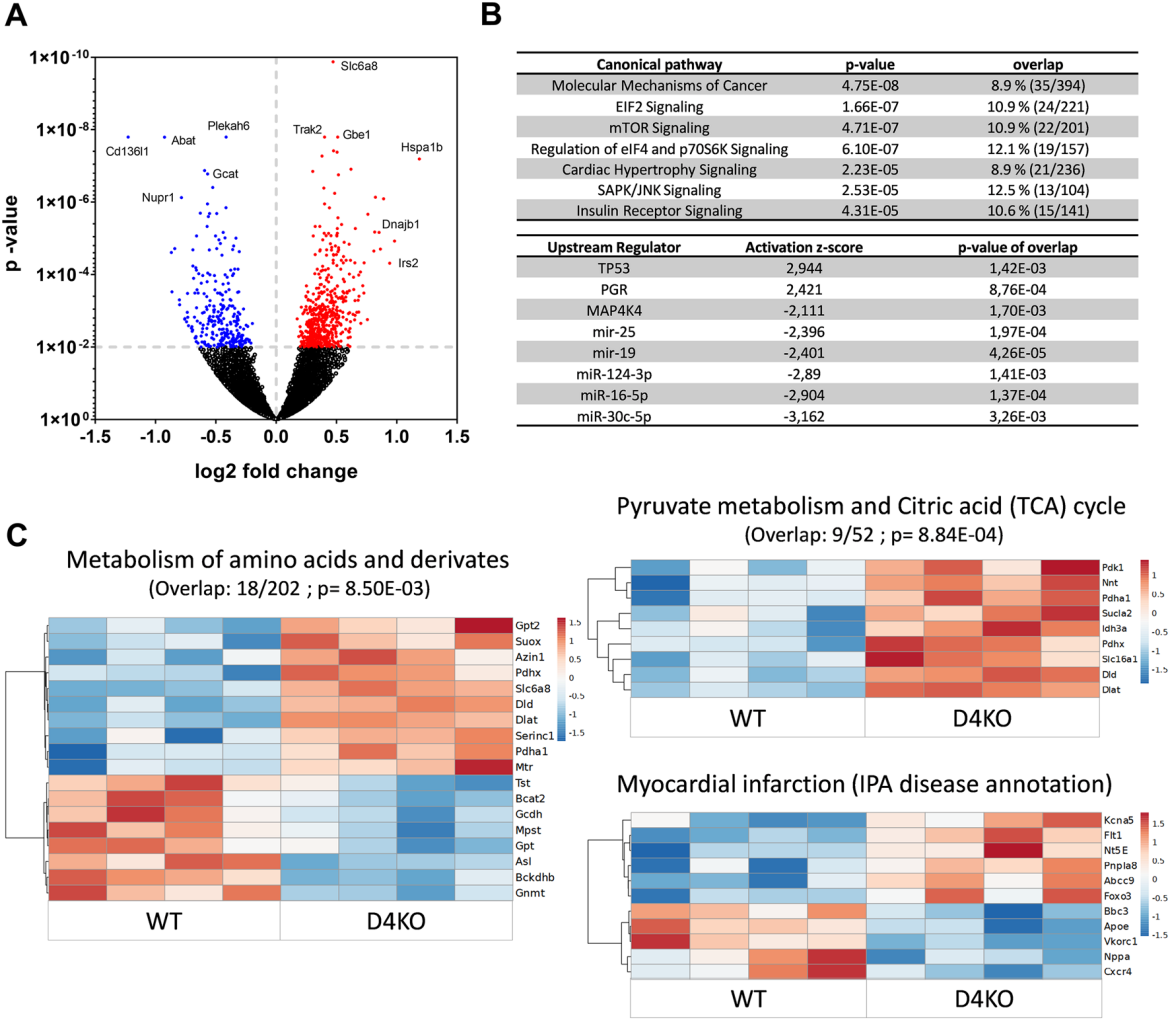
Transcriptome analysis reveals differential cardiac gene expression due to loss of *Tbc1d4*. Whole heart transcriptome analysis via RNA sequencing and subsequent Ingenuity pathway analysis **A** Volcano Plot of differentially regulated genes due to the loss of *Tbc1d4* displaying the size of differential regulation and size of significance. **B** Differentially regulated canonical pathways and potential upstream regulators of Ingenuity Pathway Analysis/IPA for differentially regulated genes with adjusted p-value < 0.01. **C** Differentially regulated genes contained in over-represented Reactome pathways and IPA disease annotations regarding altered metabolic processes. Overlap of genes and corresponding p-value of CPDB over-representation analysis are indicated above the respective heatmap Data presented as Z-Score. Significance threshold was set at p < 0.01 (n = 4). *WT* wild type, *D4KO* *Tbc1d4*-knockout

Adaptation of the heart to *Tbc1d4*-deficiency was assessed by conducting unbiased transcriptome analysis. Hearts from 36 weeks old D4KO mice and WT littermates were analyzed by RNASeq as described in the method section. We found 811 transcripts differentially expressed (537 upregulated/274 downregulated; p < 0.01) between D4KO and WT control mice (Fig. 5A). Among the differentially expressed genes, there were several highly upregulated transcripts previously associated with impaired energy metabolism and stress, such as Creatine Transporter 1 (*Slc6a8*), heat shock proteins *Hspa1, Dnaj1* and insulin receptor substrate protein 2 (*Irs2*)*,* as well as downregulated transcripts associated with amino acid degradation (*Gcat*) and response to stress signals (*Nupr1*) (Fig. 5A and Additional file 8: Table S1). Ingenuity pathway analysis (IPA) of the differentially expressed genes revealed coordinated changes in translational control and ER-stress response between the genotypes, as well as corresponding canonical pathway and upstream target candidates (Fig. 5B). Moreover, over-representation analyses revealed differential regulation of genes contained in IPA disease annotations for myocardial infarction and genes involved in Reactome pathways of amino acid and pyruvate metabolism (Fig. 5C).

**Fig. 6:**
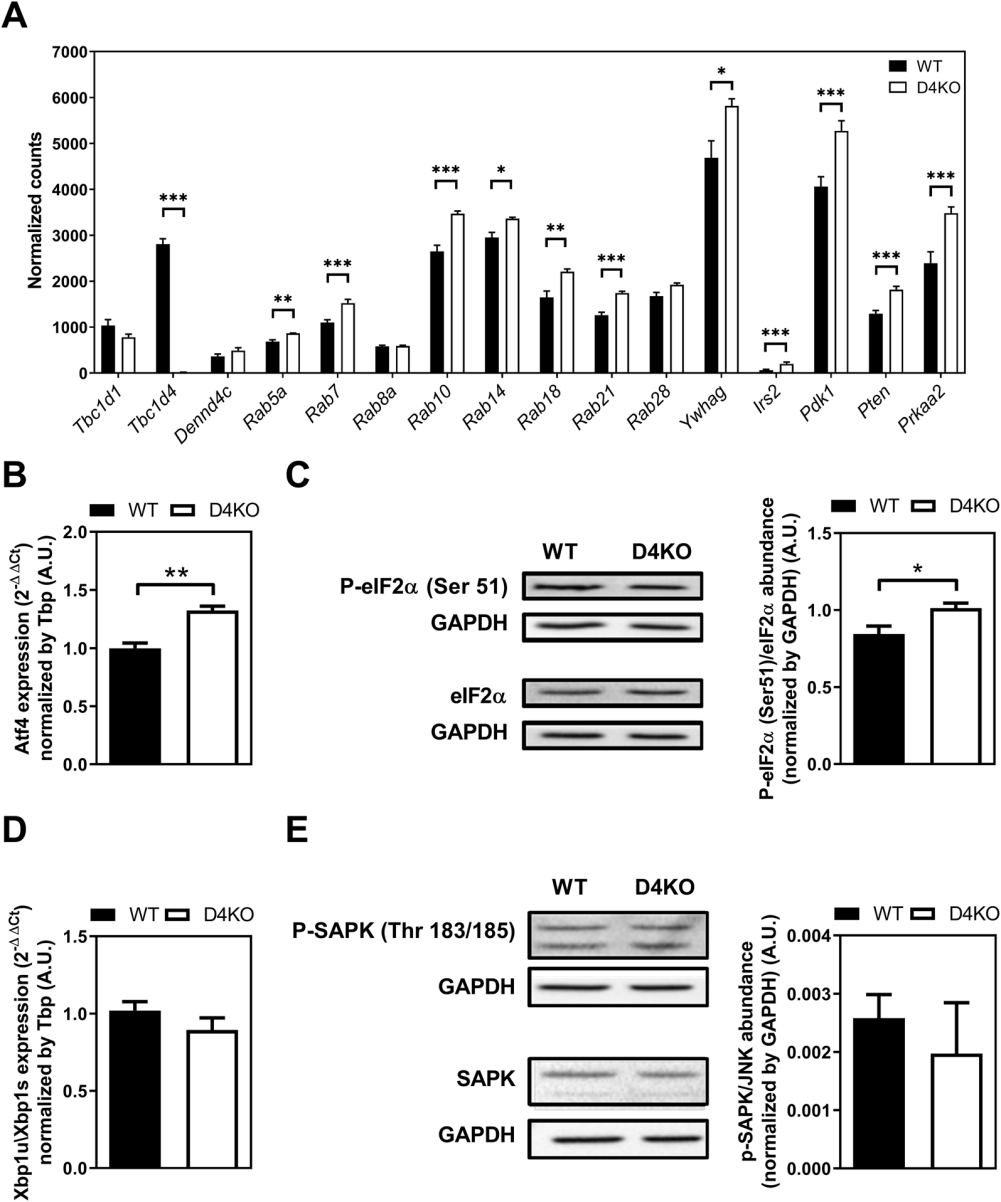
*Tbc1d4*-ko alters gene expression in insulin signaling and distinctively activates cardiac UPR via eIF2a/ATF4 pathway. **A** Expression of *Tbc1d4*-related signaling genes in D4KO and WT hearts. **B** *Atf4* expression and **C** phosphorylation ratio of phosphorylated eIF2a (Ser51) and total eIF2a protein. **D** Gene expression of *Xbp1* splicing ratio and **E** phosphorylation ratio of phosphorylated (Thr183/Tyr185) and total SAPK. Data presented as mean ± SEM. Statistical analysis was performed using unpaired two-tailed student’s t-Test (WT vs. D4KO: *p < 0.05; **p < 0.01; ***p < 0.001) n = 6–8. Male mice, 36 weeks of age. *WT* wild type, *D4KO* *Tbc1d4*-knockout

*Tbc1d4*-knockout did not alter expression of cardiac *Tbc1d1* as another RabGAP, as well as no altered expression of the counter regulatory RabGEF *Dennd4c* (Fig. 6A). However, expression levels of several RabGTPases Rab5a, Rab7, Rab10, Rab14, Rab18 and Rab21 as targets of RabGAPs were increased in D4KO hearts (Fig. 6A). In addition, transcription of genes involved in insulin signaling was also increased, including *Irs2, Ywhag (14-3-3γ), Pdk1, Pten* and the *catalytic alpha 2 subunit of AMPK*, *Prkaa2* (Fig. 6A).

We further validated differential regulation of genes involved in the ER stress response by qPCR and Western blot analysis in D4KO hearts at baseline. The mRNA expression levels of *Activating transcription factor 4* (*Atf4*) were increased in D4KO hearts compared to WT controls (Fig. 6B). In line with an increased propensity for ER stress, phosphorylation of Ser51 in eIF2α, an upstream effector of ATF4, was elevated, demonstrating enhanced eIF2α activity (Fig. 6C). Interestingly, other arms of the ER-stress response pathway remained unaffected by the knockout of *Tbc1d4*, visualized by an unaltered splicing ratio of *X-Box binding protein 1* (*Xbp1*) mRNA (Fig. 6D) as well as unaltered protein abundance and phosphorylation status of SAPK (Fig. 6E).

## Discussion

T2DM is associated with an elevated risk for CVD and poor recovery after MI. Notably, severe insulin resistance found in a subset of patients with diabetes is highly associated with cardiovascular complications [27], however, the molecular mechanisms linking insulin action and heart disease are not well understood. In the present study, we demonstrate that lack of the insulin signaling protein TBC1D4 in mice abrogates cardiac glucose uptake in response to insulin and aggravates cardiac damage after myocardial infarction. Thus, insulin mediated effects downstream of TBC1D4 are required for restoring cardiac function after MI.

The two related RabGTPases TBC1D1 and TBC1D4 are both expressed in the heart. Because in mouse skeletal muscle RabGAP expression is fiber-type specific [13, 17], we analyzed mRNA copy numbers expression levels in cardiac tissue. *Tbc1d4* mRNA had a ~ 3-times higher copy number in the left ventricle compared to *Tbc1d1,* and the most abundant isoform in the heart is the long, muscle-specific variant of the protein. Thus, cardiac RabGAP mRNA splicing and expression resembles that of adult oxidative fibers in skeletal muscle [13].

Knockout of *Tbc1d4* completely abrogated cardiac insulin-stimulated glucose uptake in vitro (LV papillary muscle) and in vivo (^18^F-FDG PET Scan) but did not affect basal glucose uptake. Despite the limitations of glucose tracers [28], our data provide direct functional evidence that TBC1D4 is required for elevated glucose uptake in response to insulin stimulation in the heart. Conversely, prior studies in TBC1D4-deficient female and male rats report increased cardiac 2-deoxyglucose uptake in vivo during a hyperinsulinemic-euglycemic clamp, despite of reduced abundance of GLUT4 and unchanged levels of glucose transporters GLUT1, GLUT8 and SGLT1 in the heart [29, 30]. Further studies are needed to investigate the subcellular localization of cardiac GLUT4 in these animals. In accordance with these studies, we also did not observe differences in expression/abundance of fatty acid transporter CD36/FAT, as well as FATP4 and FATP6, or palmitate uptake into LV papillary muscle comparing D4KO and WT mice (Additional file 6: Figure S6).

In line with reduced cardiac glucose uptake, the abundance of the glucose transporter GLUT4 in D4KO hearts was markedly reduced whereas GLUT1, the ubiquitously expressed glucose transporter, remained unchanged. Previous studies indicate that TBC1D4 deficiency results in missorting and lysosomal degradation of GLUT4 [15, 31].

Interestingly, D4KO mice had regular heart function and normal overall morphology at baseline, indicating that normal conditions reduced insulin-stimulated glucose uptake in the heart may not essentially affect the maintenance of heart functions. However, complete ablation of GLUT4, either whole-body or heart-specific knockout, has been shown to result in cardiac hypertrophy and premature death [32, 33]. Moreover, lack of GLUT4 has been associated with reduced glycolysis, increased glycogen stores, accelerated ATP depletion during ischemia and lower phosphocreatine (PCr) after I/R in the heart [34]. The elevated glycogen levels in *Tbc1d4*-deficient hearts may result from complex counterregulation of glycogen synthesis and breakdown as observed in muscle-specific *Glut4*-knockout mice [35]. As GLUT4 constitutes the most abundant glucose transporter in the heart and translocates from intracellular vesicles to the plasma membrane in response to insulin, ischemia, and hypoxia, it may provide myocardial protection during ischemia [36]. Hence, the role of GLUT4 in cardiac energy metabolism might be not of utmost importance under non-stressed conditions, but becomes obligate in the adaptation under conditions of hemodynamic stress [33]. Interestingly, GLUT4 knockout abrogates insulin-stimulated glucose uptake in the heart but leads to a compensatory increase in GLUT1, associated with substantially elevated basal glucose uptake [32]. In fact, the cardiac dysfunction of GLUT4-deficient mice has been attributed to an increase in basal GLUT1-mediated glucose uptake in [32]. Therefore, D4KO mice exhibit a unique cardiac phenotype with severely compromised insulin-stimulated glucose transport but apparently normal glucose flux and heart function in the basal state. Furthermore, our data indicate that lack of insulin-stimulated glucose transport is not sufficient to trigger a compensatory increase in cardiac expression of GLUT1.

I/R-induced myocardial injury was more pronounced in D4KO mice compared to WT littermates with knockout mice showing progressive impairment in cardiac function and a ~ 30% higher infarction size 3 weeks *post* surgery, indicating that TBC1D4 is required for the *post* infarction healing processes. Further studies are needed to determine whether the decrease in wall thickness results from alteration in size/number of myocytes as well and/or changes in the extracellular matrix. A recent study investigated cardiac phenotypes in *Tbc1d4*^T649A^ knockin mice that lack a major AKT phosphorylation site. Heart function of *Tbc1d4*^T649A^ mice under basal and infarct conditions were not different, and infarction areas were similar compared to control mice, whereas the related *Tbc1d1* was upregulated in the hearts of knockin mice [37]. Nevertheless, the study used a different infarction protocol with a permanent occlusion of the LAD. Moreover, the *Tbc1d4*^T649A^ mutation may not result in a complete loss-of-function of the RabGAP, and its impact on cardiac glucose metabolism and GLUT4 content remains to be determined. Interestingly, *Tbc1d4*^T649A^ mice displayed increased R-wave amplitudes at baseline, whereas our study also found respective increases during ischemia and reperfusion. These changes may indicate structural alterations in the myocardium in response to impaired RabGAP signaling that impair electrical conductivity of the heart in the absence of major cardiac dysfunction.

Unexpectedly, *Tbc1d4* deficiency was associated with a marked increase in cardiac ECM area, as revealed by TEM imaging and morphometry. In line, biochemical analysis revealed impaired expression of MMPs and altered ratios of MMPs and their corresponding TIMPs that are essential for normal ECM remodeling and function [38–40]. Increased ECM mass has been associated with cardiac fibrosis, myocardial stiffness and cardiac dysfunction [41] and thus may contribute to the impaired recovery of D4KO hearts after I/R. While the mechanistic link of cardiac ECM dynamics and TBC1D4 is unclear, it is important to note that secretion of MMPs has been found to be regulated by Rab GTPases [42, 43], suggesting a relation of MMP action and vesicular traffic. Interestingly, MMP14 exocytosis and secretion has been shown to be dependent on a subset of Rabs, including *Rab5a*, *Rab8* and *Rab14* [44, 45], the latter two being substrates for TBC1D4 [46]. Interestingly, the expression of *Rab5a* and *Rab14* was higher in *Tbc1d4*-knockout hearts compared to WT controls. However, further studies are required to elucidate the role of RabGAP signaling in ECM remodeling and direct effects on the I/R-induced myocardial damage. However, an altered ECM remodeling and MMP/TIMP expression in the basal state might contribute to impaired recovery after I/R. MMPs have been shown to be active in cardiac post-MI remodeling and alterations of MMP levels may lead to impairments of heart structure and heart functions [47]. Moreover, upregulation of genes involved in insulin- and related signaling such as *Irs2, Pdk1, Pten and Prkaa2* indicate that dysregulation already at baseline may contribute to a worsened phenotype of D4KO hearts following I/R through impaired metabolic signaling.

Transcriptome analysis of hearts from D4KO mice and WT littermates revealed differential expression of genes associated with cardiac hypertrophy, cellular metabolic stress response and cell survival prior to the I/R intervention and in the absence of impaired heart function. Among the most significantly upregulated genes was the creatine transporter *Slc6a8* which is critical for cardiac ATP generation [48]. Interestingly, cardiac creatine (Cr) content was highly elevated in the heart of GLUT4 knockout mice, presumably to compensate for reduced glucose-derived energy formation [34]. Ablation of *Tbc1d4* also impacts transcription of genes in canonical pathways annotated for CVD-associated phenotypes (cardiac hypertrophy signaling) prior to I/R intervention. This could indicate a subclinical imbalance of cardiac and diabetes-associated signaling cascades, which would be triggered during pathological events like I/R. Thus, our data suggest that hearts from D4KO have compromised metabolic flexibility, which may translate into reduced myocardial protection against ischemia and reperfusion injury.

Among the differentially expressed transcripts were markers for cardiac unfolded protein response of the eIF2a/ATF4 pathway. ATF4 as a transcription factor has been reported to regulate various stress genes involved in cardiomyocytes death [49]. On the other hand, ATF4 action is important in order to restore ER homeostasis in cardiomyocytes following ischemia [50]. While the molecular role of *Tbc1d4* on the mediation of the ER-Stress response remains to be elucidated, it is tempting to speculate that alterations in vesicle trafficking due to reduced RabGAP activity may explain changes in organelle integrity and homeostasis.

A common nonsense mutation in *TBC1D4* (Arg684Ter) was recently identified in Greenlandic Inuit and other arctic populations and has been associated with severe glucose intolerance, reduced GLUT4 abundance in skeletal muscle and increased risk for T2DM [18]. Interestingly, this mutation maps to a muscle-specific exon in the *TBC1D4* gene, rendering homozygous carriers of the allele knockouts in skeletal muscle [18, 19]. In this study we show that murine cardiac muscle exclusively contains the long isoform of *Tbc1d4*, suggesting that the human homozygous Arg684Ter allele carriers may also lack TBC1D4 in the heart. A recent study found an increased incidence of CVD and CVD deaths in a Greenlandic sample but without reaching statistical significance [51]. Unexpectedly, T2DM was also not associated with an elevated risk for CVD in that study, possibly indicating a lack of sufficient power to link the Arg684Ter variant with cardiovascular related traits. Thus, the role of TBC1D4 deficiency in T2DM, and the contribution of systemic insulin resistance to the cardiac phenotype remains to be further investigated.

Collectively, our study has identified TBC1D4 as an essential component for cardiac glucose uptake and a determinant in the response to cardiac I/R-induced injury. While RabGAPs might be suitable targets for therapeutic interventions, further return-of-function studies need to be conducted to prove possible modulation of insulin-responsive glucose transport. Specifically, increasing TBC1D4 activity might improve cardiac glucose utilization and protect from myocardial damage in response to MI.

## Supplementary Information

**Additional file 1: Figure S1.** Expression of *Tbc1d4* isoforms in different murine tissues. At 555 bp Tbc1d4 isoform lacking exon 10 and at 782bp Tbc1d4 variant including exon 10. WAT = white adipose tissue, BAT = brown adipose tissue, SM= skeletal muscle.

**Additional file 2: Figure S2.** Feeding of a 60% high-fat diet leads to impaired whole-body glucose tolerance in C57BL/6J mice. Blood glucose concentrations in (A) Chow-fed and (B) HFD-fed male C57BL6/J mice at 34-37 weeks of age after 6h of fasting and subsequent intraperitoneal injection of glucose (2 mg/kg). Data presented as mean ± SEM. n=23-28; HFD=high-fat diet, WT=wild type, D4KO=Tbc1d4-knockout.

**Additional file 3: Figure S3.** Echocardiographic assessment of mouse heart function/morphology after I/R. Following the I/R-intervention phase (closed chest), animals were monitored for the 3 week reperfusion phase. During this, parameters of cardiac function and morphology were measured before the intervention (baseline) and at time points of 24 hours, 1 week and 3 weeks after the intervention for type (WT; white) and *Tbc1d4*- deficient (D4KO; grey). AoV = aortic valve, VTI = velocity time integral, MV = mitral valve, IVS = intraventricular septum, LVPW = left ventricular posterior wall, LVID = left ventricular inner diameter, LV = left ventricle. Data are presented as mean values ± SEM (n = 6). Two-tailed unpaired Student´s t-test with Welch´s correction.

**Additional file 4: Figure S4.** PET scan analysis of in vivo [^18^F]-FDG glucose uptake into the heart within the initial 5 minutes after injection. This data is equivalent to the one shown in Figure 2, but re-analyzed for the first 5 minutes after injection. (A) Quantification of cardiac glucose uptake and (B) corresponding area under the curve (AUC) calculation. Data are presented as mean values ± SEM (n=4). Two-tailed unpaired Student´s t-test with Welch´s correction (**p<0.01).

**Additional file 5: Figure S5.** PET scan analysis of in vivo [^18^F]-FDG glucose uptake into the brain. (A) Quantification of glucose uptake into the brain over time and (B) corresponding area under the curve (AUC) calculation. Data are presented as mean values ± SEM (n=4). Two-tailed unpaired Student´s t-test with Welch´s correction.

**Additional file 6: Figure S6.** *Tbc1d4*-knockout has no effect on palmitate uptake into LV papillary muscle and whole heart fatty acid transporter expression/abundance. (A) [^3^H]Palmitate uptake into isolated left ventricular papillary muscle. Briefly, animals were fasted for 16h before isolation and transfer of LV papillary muscle into Krebs-Henseleit buffer (supplemented with glucose, mannitol and fatty acid-free BSA for 15 min. Subsequently, muscles were incubated for 2h under presence of tritiated palmitate. Muscles were removed, homogenized and centrifuged. Cleared supernatant was used for scintillation counting and protein content quantification. (B-D) Whole heart expression of the fatty acid transporters *Cd36* (Fwd: GATGTGCAAAACCCAGATGA; Rev: TCCTCGGGGTCCTGAGTTAT), *Slc27a4* (Fwd: CGCTGGAAAGGGGAGAATGT; Rev: AGTTCCTGGCACCTCAACAC) and *Slc27a6* (Fwd: TCGGAAGGGAGACGTGTACT; Rev: TCATAACCTGGCACACGC) was determined via qPCR and according protein abundance of (E-G) CD36/FAT, FATP4 and FATP6 was assessed vie Western Blotting. LV = left ventricle. Data are presented as mean values ± SEM (n=4-8). Two-tailed unpaired Student´s t-test with Welch´s correction.

**Additional file 7: Figure S7.** Representative M-mode echocardiography images of *Tbc1d4*-ko and WT mice. WT=wild type, D4KO=*Tbc1d4*-knockout.

**Additional file 8: Table S1. **TOP 25 up- and downregulated genes of cardiac transcriptome due to *Tbc1d4*-knockout.** Table S2.** Primary antibody suppliers and Western Blotting concentrations.

## Data Availability

All the data supporting the findings of this study are available from the corresponding author upon reasonable request. RNA-sequencing data have been deposited in the BioProject/SRA database with the accession “PRJNA830326” (https://www.ncbi.nlm.nih.gov/bioproject/?term=PRJNA830326) and will be available upon acceptance of the manuscript. H. A-H. and A.C. had full access to all the data in the study and takes responsibility for its integrity and the data analysis.

## Abbreviations

T2DM: Type 2 diabetes mellitus
MI: Myocardial infarction
I/R: Ischemia/reperfusion
ER: Endoplasmic reticulum
ECM: Extracellular matrix
LV: Left ventricle
D4KO: *Tbc1d4*-knockout
