# Supplementary figures and images for: Deletion of Tbc1d4/As160 abrogates cardiac glucose uptake and increases myocardial damage after ischemia/reperfusion
Source: Cardiovasc Diabetol. 2023 Jan 27;22:17. doi: 10.1186/s12933-023-01746-2 (PMC9881301; doi:10.1186/s12933-023-01746-2)

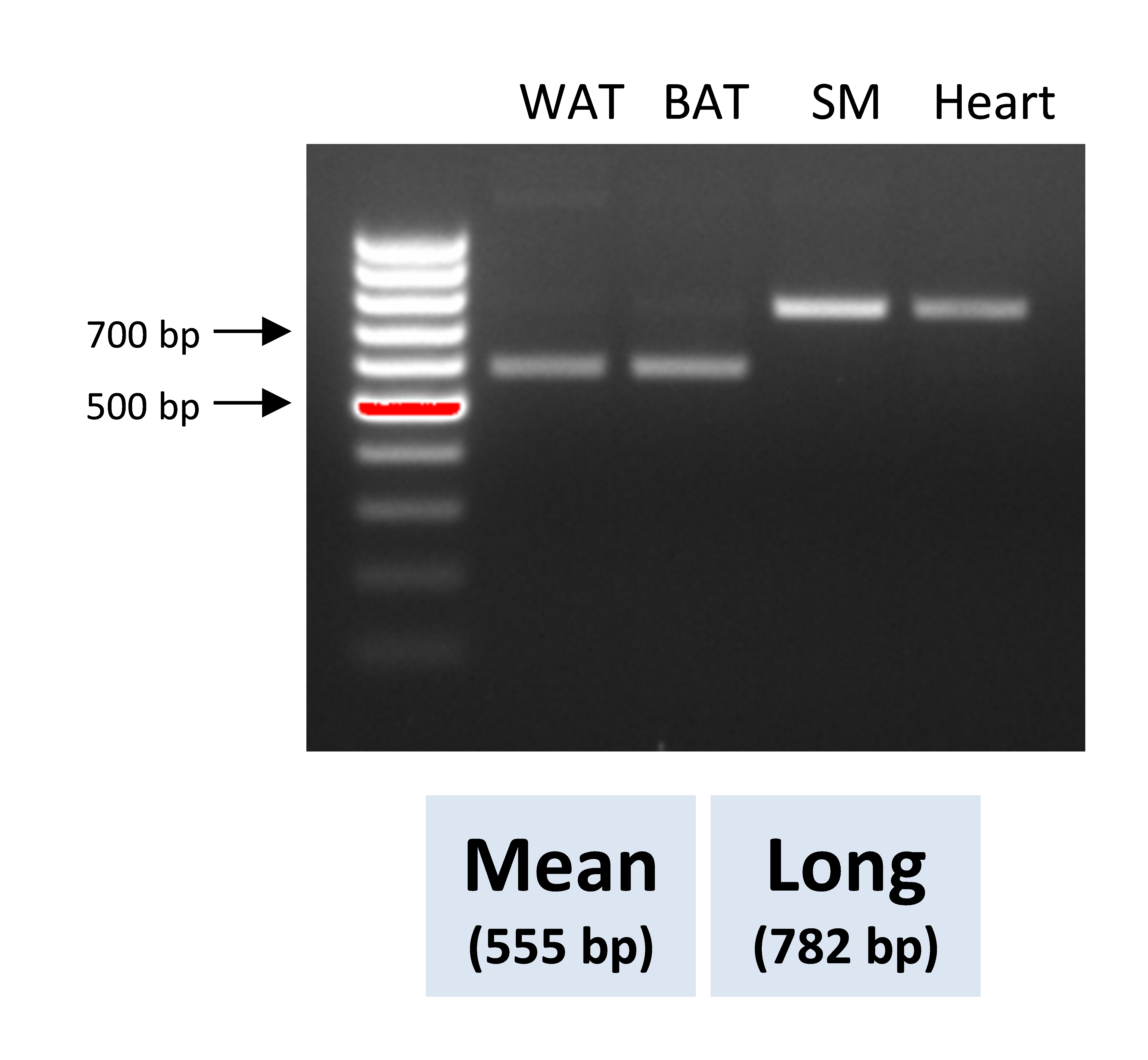

Supplement: Supplementary file 1 — Additional file 1: Figure S1. Expression of Tbc1d4 isoforms in different murine tissues. At 555 bp Tbc1d4 isoform lacking exon 10 and at 782bp Tbc1d4 variant including exon 10. WAT = white adipose tissue, BAT = brown adipose tissue, SM= skeletal muscle. [file 12933_2023_1746_MOESM1_ESM.tif]

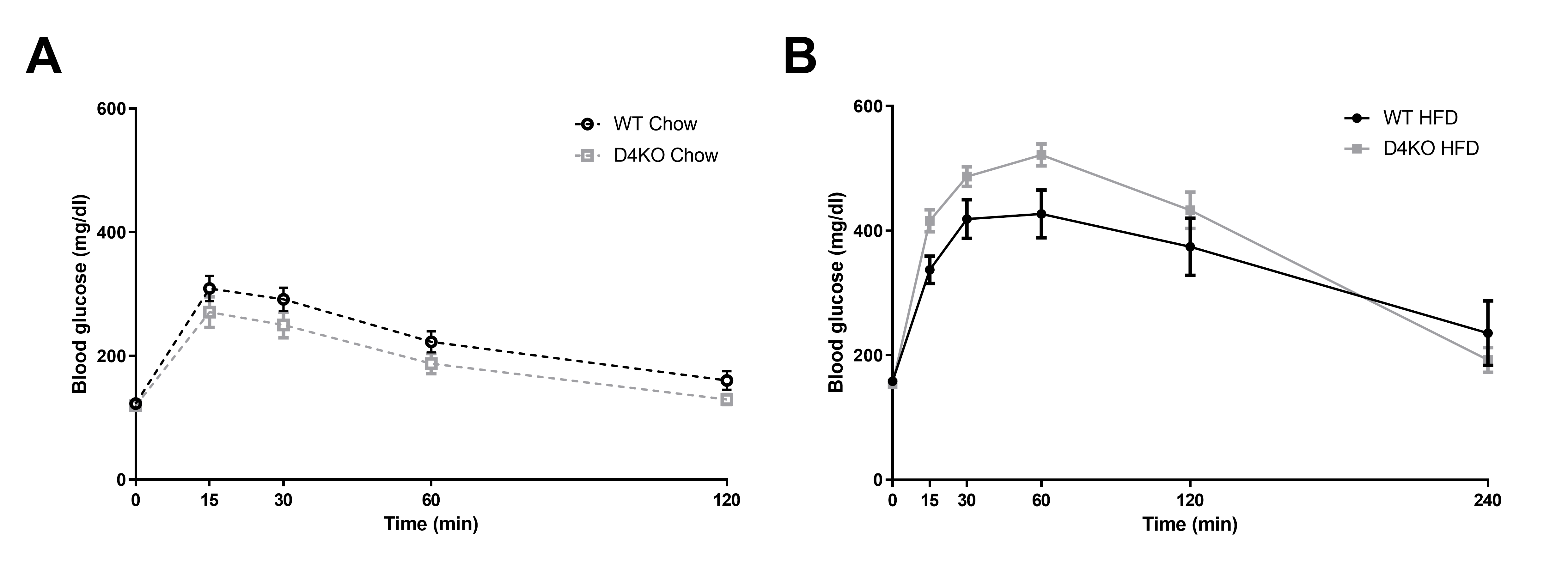

Supplement: Supplementary file 2 — Additional file 2: Figure S2. Feeding of a 60% high-fat diet leads to impaired whole-body glucose tolerance in C57BL/6J mice. Blood glucose concentrations in (A) Chow-fed and (B) HFD-fed male C57BL6/J mice at 34-37 weeks of age after 6h of fasting and subsequent intraperitoneal injection of glucose (2 mg/kg). Data presented as mean ± SEM. n=23-28; HFD=high-fat diet, WT=wild type, D4KO=Tbc1d4-knockout. [file 12933_2023_1746_MOESM2_ESM.tif]

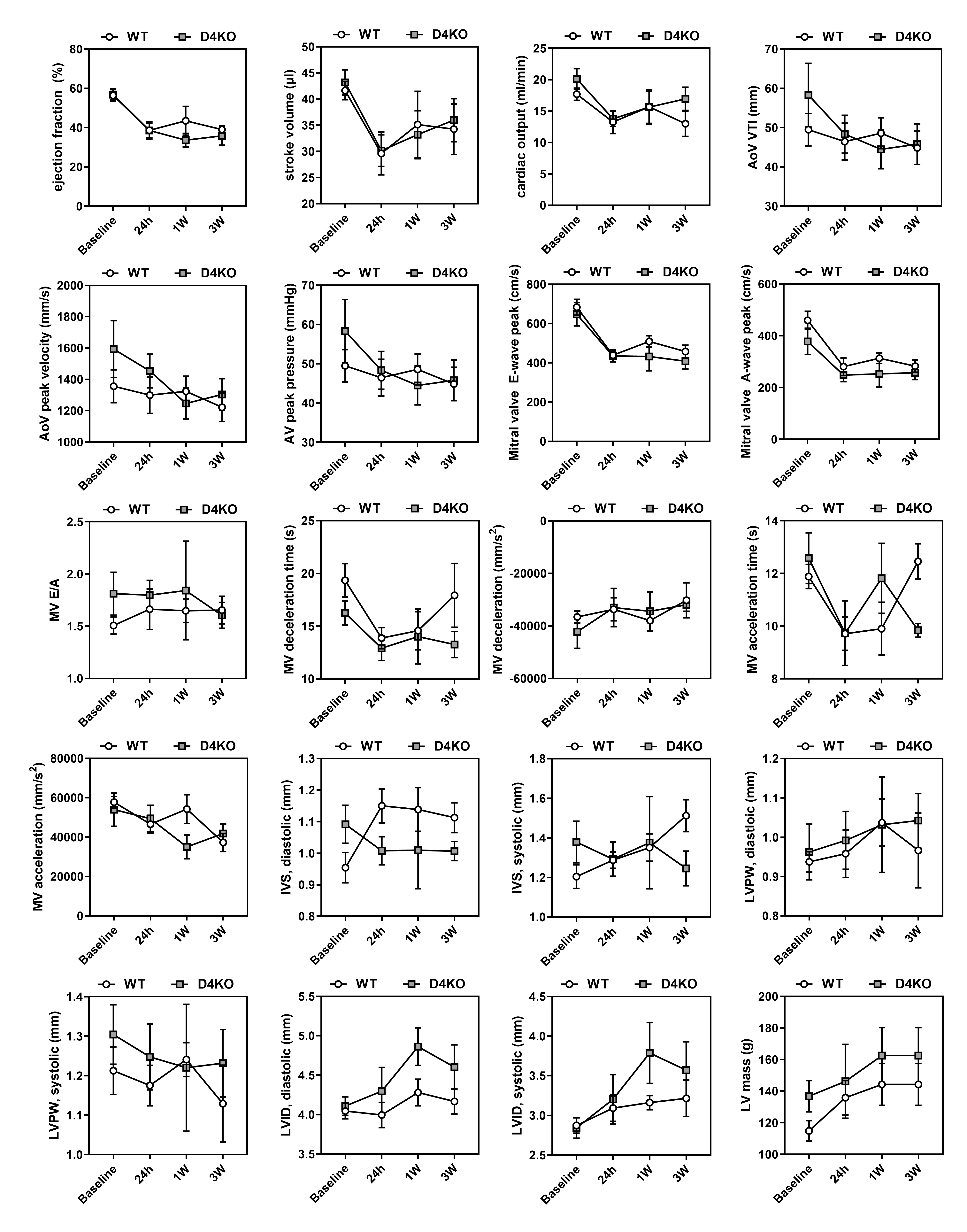

Supplement: Supplementary file 3 — Additional file 3: Figure S3. Echocardiographic assessment of mouse heart function/morphology after I/R. Following the I/R-intervention phase (closed chest), animals were monitored for the 3 week reperfusion phase. During this, parameters of cardiac function and morphology were measured before the intervention (baseline) and at time points of 24 hours, 1 week and 3 weeks after the intervention for type (WT; white) and Tbc1d4- deficient (D4KO; grey). AoV = aortic valve, VTI = velocity time integral, MV = mitral valve, IVS = intraventricular septum, LVPW = left ventricular posterior wall, LVID = left ventricular inner diameter, LV = left ventricle. Data are presented as mean values ± SEM (n = 6). Two-tailed unpaired Student´s t-test with Welch´s correction. [file 12933_2023_1746_MOESM3_ESM.tif]

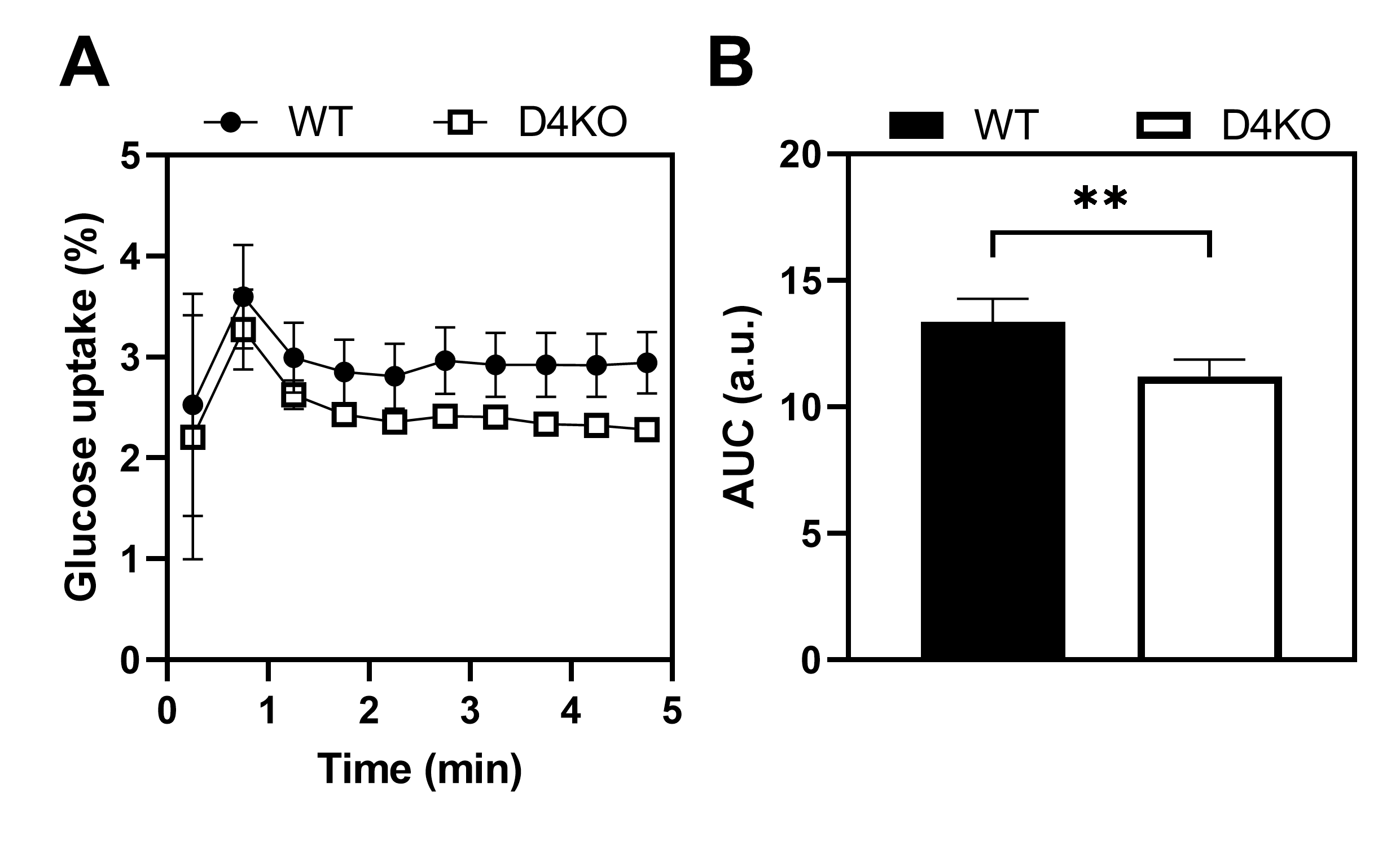

Supplement: Supplementary file 4 — Additional file 4: Figure S4. PET scan analysis of in vivo [18F]-FDG glucose uptake into the heart within the initial 5 minutes after injection. This data is equivalent to the one shown in Figure 2, but re-analyzed for the first 5 minutes after injection. (A) Quantification of cardiac glucose uptake and (B) corresponding area under the curve (AUC) calculation. Data are presented as mean values ± SEM (n=4). Two-tailed unpaired Student´s t-test with Welch´s correction (**p<0.01). [file 12933_2023_1746_MOESM4_ESM.tif]

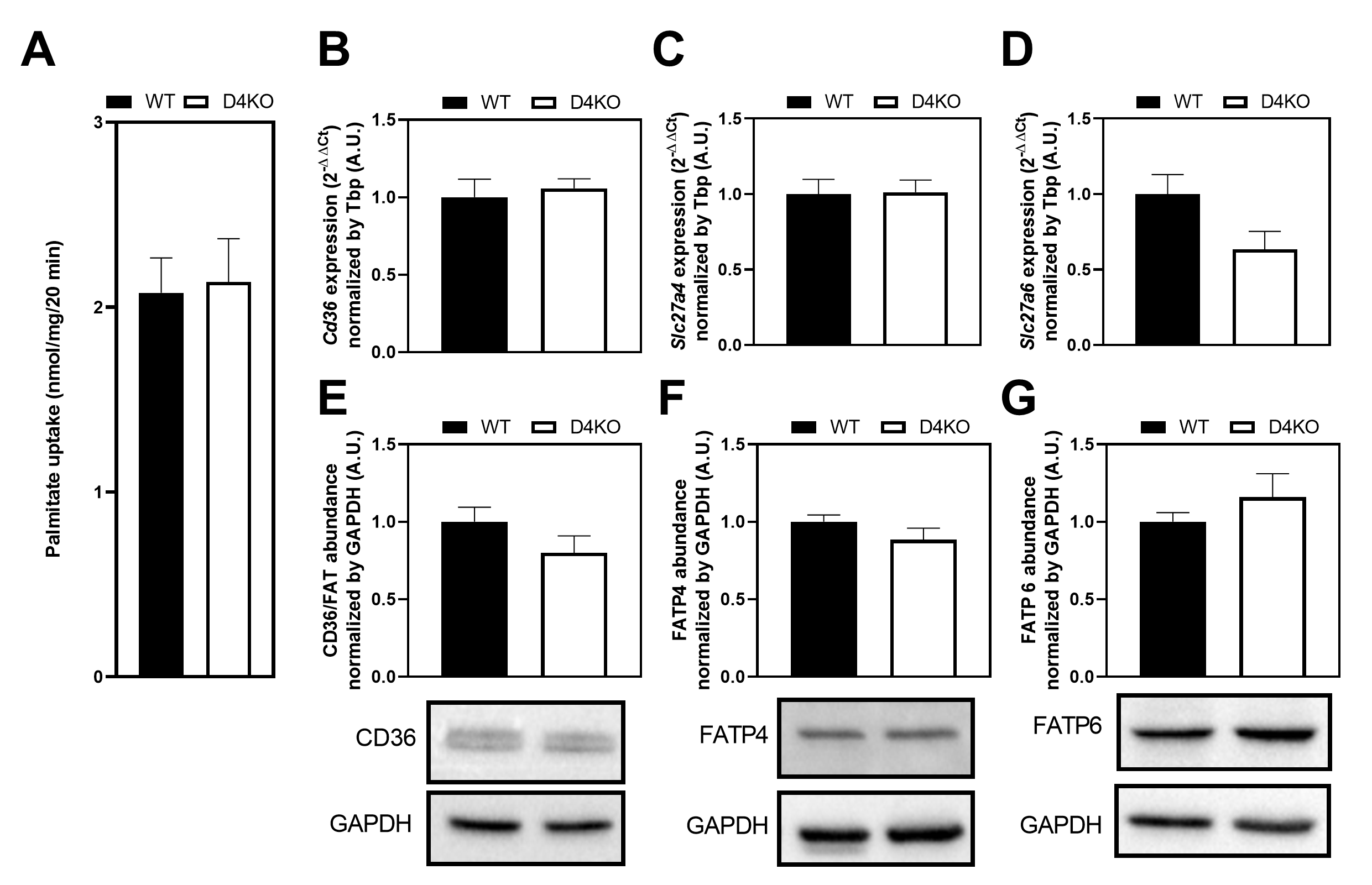

Supplement: Supplementary file 5 — Additional file 5: Figure S5. PET scan analysis of in vivo [18F]-FDG glucose uptake into the brain. (A) Quantification of glucose uptake into the brain over time and (B) corresponding area under the curve (AUC) calculation. Data are presented as mean values ± SEM (n=4). Two-tailed unpaired Student´s t-test with Welch´s correction. [file 12933_2023_1746_MOESM5_ESM.tif]

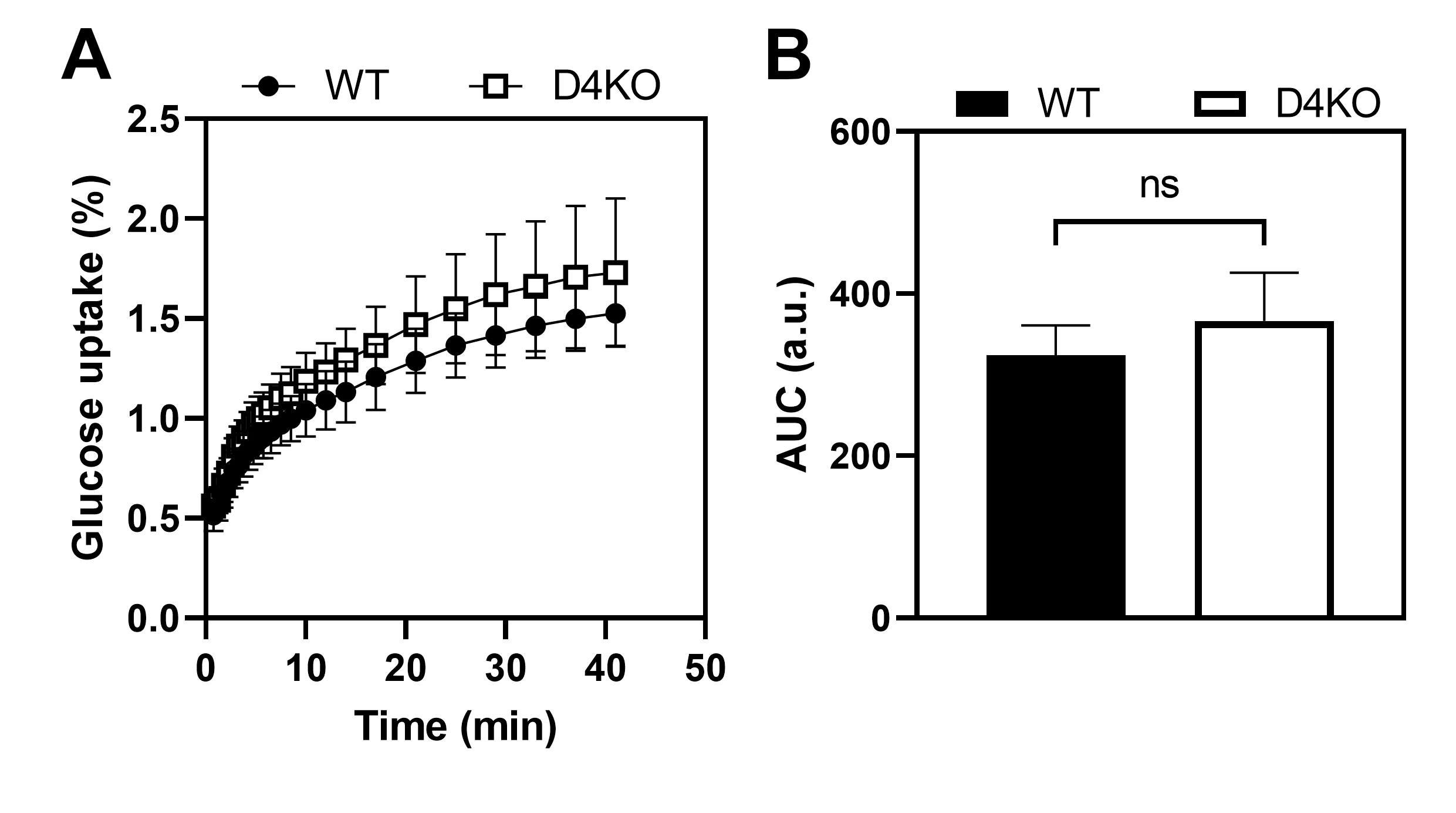

Supplement: Supplementary file 6 — Additional file 6: Figure S6. Tbc1d4-knockout has no effect on palmitate uptake into LV papillary muscle and whole heart fatty acid transporter expression/abundance. (A) [3H]Palmitate uptake into isolated left ventricular papillary muscle. Briefly, animals were fasted for 16h before isolation and transfer of LV papillary muscle into Krebs-Henseleit buffer (supplemented with glucose, mannitol and fatty acid-free BSA for 15 min. Subsequently, muscles were incubated for 2h under presence of tritiated palmitate. Muscles were removed, homogenized and centrifuged. Cleared supernatant was used for scintillation counting and protein content quantification. (B-D) Whole heart expression of the fatty acid transporters Cd36 (Fwd: GATGTGCAAAACCCAGATGA; Rev: TCCTCGGGGTCCTGAGTTAT), Slc27a4 (Fwd: CGCTGGAAAGGGGAGAATGT; Rev: AGTTCCTGGCACCTCAACAC) and Slc27a6 (Fwd: TCGGAAGGGAGACGTGTACT; Rev: TCATAACCTGGCACACGC) was determined via qPCR and according protein abundance of (E-G) CD36/FAT, FATP4 and FATP6 was assessed vie Western Blotting. LV = left ventricle. Data are presented as mean values ± SEM (n=4-8). Two-tailed unpaired Student´s t-test with Welch´s correction. [file 12933_2023_1746_MOESM6_ESM.tif]

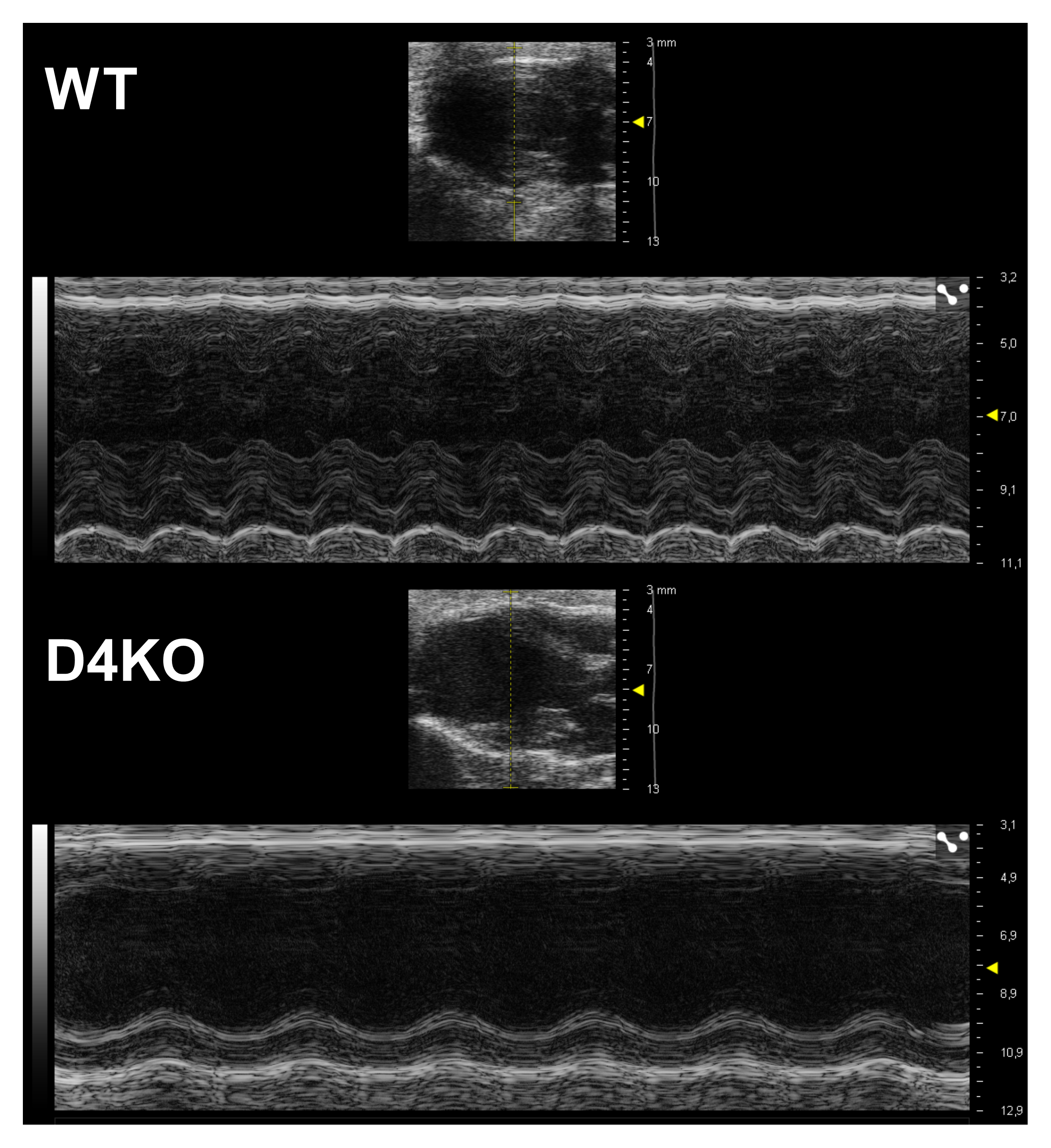

Supplement: Supplementary file 7 — Additional file 7: Figure S7. Representative M-mode echocardiography images of Tbc1d4-ko and WT mice. WT=wild type, D4KO=Tbc1d4-knockout. [file 12933_2023_1746_MOESM7_ESM.tif]
