# Supplementary material for: Deletion of Tbc1d4/As160 abrogates cardiac glucose uptake and increases myocardial damage after ischemia/reperfusion
Source: Cardiovasc Diabetol. 2023 Jan 27;22:17. doi: 10.1186/s12933-023-01746-2 (PMC9881301; doi:10.1186/s12933-023-01746-2)
Supplement: Supplementary file 8 — Additional file 8: Table S1. TOP 25 up- and downregulated genes of cardiac transcriptome due to Tbc1d4-knockout. Table S2. Primary antibody suppliers and Western Blotting concentrations. [file 12933_2023_1746_MOESM8_ESM.docx]

**Supplementary table S1: TOP 25 up- and downregulated genes of cardiac transcriptome due to *Tbc1d4*-knockout.**

| **TOP 25 upregulated genes**  **(D4KO vs WT)** | | | | **TOP 25 downregulated genes**  **(D4KO vs WT)** | | |
| --- | --- | --- | --- | --- | --- | --- |
| *gene* | *log2 fold change* | *p-value* | *gene* | | *log2 fold change* | *p-value* |
| *Slc6a8* | 0,472 | 1,33E-10 | *Tbc1d4* | | -6,129 | 0 |
| *Trak2* | 0,401 | 1,59E-08 | *Cd163l1* | | -1,228 | 1,59E-08 |
| *Gbe1* | 0,510 | 1,59E-08 | *Plekha6* | | -0,415 | 1,59E-08 |
| *Coa5* | 0,476 | 3,82E-08 | *Abat* | | -0,925 | 1,60E-08 |
| *Pptc7* | 0,504 | 4,16E-08 | *2410002F23Rik* | | -0,594 | 1,34E-07 |
| *Nek9* | 0,380 | 5,30E-08 | *Gcat* | | -0,568 | 1,65E-07 |
| *Hspa1b* | 1,186 | 6,38E-08 | *Klhdc8b* | | -0,526 | 3,92E-07 |
| *Commd6* | 0,620 | 1,23E-07 | *Nupr1* | | -0,786 | 7,47E-07 |
| *Dlat* | 0,303 | 1,41E-07 | *Plcd1* | | -0,570 | 1,11E-06 |
| *Akap2* | 0,516 | 1,76E-07 | *Mical1* | | -0,416 | 1,41E-06 |
| *Pcyt1a* | 0,395 | 4,08E-07 | *Tkt* | | -0,629 | 2,01E-06 |
| *Golga4* | 0,485 | 5,70E-07 | *Adamts10* | | -0,552 | 2,06E-06 |
| *Frmd5* | 0,822 | 7,25E-07 | *Mum1* | | -0,493 | 2,06E-06 |
| *Errfi1* | 0,890 | 8,04E-07 | *Abhd14b* | | -0,562 | 2,49E-06 |
| *Slc25a13* | 0,401 | 1,11E-06 | *Pdlim2* | | -0,573 | 4,66E-06 |
| *Akap1* | 0,441 | 1,43E-06 | *Xpo6* | | -0,439 | 6,84E-06 |
| *Ell2* | 0,761 | 2,16E-06 | *Csrp2* | | -0,660 | 8,52E-06 |
| *Klf6* | 0,512 | 2,70E-06 | *Meg3* | | -0,503 | 8,52E-06 |
| *Tead1* | 0,542 | 4,11E-06 | *Slc50a1* | | -0,425 | 9,67E-06 |
| *Slc25a12* | 0,324 | 4,66E-06 | *Mapk12* | | -0,333 | 9,80E-06 |
| *Dld* | 0,361 | 4,66E-06 | *Gstm2* | | -0,475 | 1,09E-05 |
| *Cpox* | 0,507 | 5,30E-06 | *Whamm* | | -0,462 | 1,38E-05 |
| *Dnaja1* | 0,656 | 5,30E-06 | *Rpl13a* | | -0,353 | 1,61E-05 |
| *Ammecr1* | 0,817 | 6,67E-06 | *Per3* | | -0,841 | 1,91E-05 |
| *Dnajb1* | 0,853 | 2,81E-03 | *Iffo1* | | -0,493 | 1,91E-05 |

Cardiac transcriptome of wild type (WT) and *Tbc1d4*-deficient (D4KO) hearts from 3 weeks post-I/R samples were sequenced using RNASequencing and subsequently tested for differential gene expression. Significance threshold was set at an adjusted p-value of p<0.01 (n=4).

**Supplementary Table S2: Primary antibody suppliers and Western Blotting concentrations.**

| **Target protein** | **Supplier** | **Concentration** |
| --- | --- | --- |
| GLUT1 | Generous gift from Dr. Anette Schürmann (German Institute of Human Nutrition, Potsdam, Germany) | 1:1,000 (in 5% skim milk/1xTBST) |
| GLUT4 | Custom made (Dr. Hadi Al-Hasani) | 1:1,000 (in 5% skim milk/1xTBST) |
| GAPDH | Cell Signaling (#2118), Denvers, USA | 1:5,000 (in 5% BSA/1xTBST) |
| eIF2α | Cell Signaling (#9722), Denvers, USA | 1:2,500 (in 5% BSA/1xTBST) |
| Phospho- eIF2α (Ser51) | Cell Signaling (#9721), Denvers, USA | 1:1,000 (in 5% skim milk/1xTBST) |
| SAPK/JNK | Cell Signaling (#9252), Denvers, USA | 1:1,000 (in 5% skim milk/1xTBST) |
| Phospho-SAPK/JNK (Thr183/Tyr185) | Cell Signaling (#4668), Denvers, USA | 1:1,000(in 5% skim milk/1xTBST) |
| CD36/FAT | R&D systems (MAB2519), Minneapolis, USA | 1:1,000 (in 5% skim milk/1xTBST) |
| FATP4 | Santa Cruz (sc-25670), Dallas, USA | 1:1,000 (in 5% skim milk/1xTBST) |
| FATP6 | Abnova (PAB27421), Taipei City, Taiwan | 1:1,000 (in 5% skim milk/1xTBST) |
